# Is biomass fuel smoke exposure associated with anemia in non-pregnant reproductive-aged women?

**DOI:** 10.1371/journal.pone.0272641

**Published:** 2022-08-19

**Authors:** Malshani Lakshika Pathirathna, Buddhini Piumi Pabasara. Samarasekara, Charitha Mendis, Chandraratne Mahinda Bandara Dematawewa, Kayako Sekijima, Mieko Sadakata, Yoshiyuki Muramatsu, Naoshi Fujiwara

**Affiliations:** 1 Faculty of Allied Health Sciences, Department of Nursing, University of Peradeniya, Peradeniya, Sri Lanka; 2 Faculty of Allied Health Sciences, Department of Medical Laboratory Science, University of Peradeniya, Peradeniya, Sri Lanka; 3 Faculty of Agriculture, Department of Animal Science, University of Peradeniya, Sri Lanka; 4 Graduate School of Health Sciences, Niigata University, Asahimachi-dori, Chuo-ku, Niigata, Japan; James Cook University, AUSTRALIA

## Abstract

**Objectives:**

Sri Lanka is a developing country where the majority of households still rely on firewood for cooking. Furthermore, the prevalence of anemia among reproductive-aged women is of moderate public health importance, according the classification of World Health Organization. Despite the researchers’ ongoing efforts to investigate a link between solid fuel smoke exposure and anemia, the veracity of their findings remains uncertain. As a result, the purpose of this study was to examine the relationship between biomass fuel smoke exposure and anemia in non-pregnant reproductive-aged women in Sri Lanka.

**Methods:**

A descriptive cross-sectional study was conducted among 382 non-pregnant reproductive-aged (15 to 49 years) women in Central Province, Sri Lanka. Data was collected using a standardized interviewer-administered questionnaire, and exposure was assessed using a breath carbon monoxide monitor. Drabkin’s cynomethhemoglobin technique was used to determine blood hemoglobin concentration.

**Results:**

The overall prevalence of anemia was 36.1%. The logistic regression model revealed no effect of cooking fuel type on anemic or non-anemic status after adjusting for potential confounding factors (p > 0.05). The multivariate regression analysis also discovered that cooking fuel type had no effect on women’s blood hemoglobin concentration.

**Conclusions:**

The study results suggest no impact of solid fuel smoke exposure on anemia among non-pregnant, reproductive-aged women. Larger scale prospective cohort studies are recommended. The reasons behind the high prevalence of anemia among reproductive-aged women should be further investigated, and corrective measures should be implemented urgently.

## Introduction

Anemia is a condition when the quantity of red blood cells or the hemoglobin concentration is lower than usual. The delivery of oxygen necessitates the presence of hemoglobin and the capacity of the blood to deliver oxygen to the body’s tissues is lowered if there are too few or defective red blood cells or not enough hemoglobin [1]. The global prevalence of anemia among non-pregnant women of reproductive age was 29.6% in 2019 [2]. Lower-middle-income nations had the highest prevalence (43.7%) of anemia among reproductive-aged women, followed by low-income countries with the second-highest incidence (38.8%) [2]. Sri Lanka is a country in the Southeast Asian region that upgraded to a lower-middle-income status recently. In 2019, the reported prevalence of anemia among non-pregnant reproductive-aged women in Sri Lanka was 34.6%, the second-highest prevalence among the countries in the Southeast Asian region, second only to India’s rates [2]. Anemia is a multifactorial condition caused by excessive iron demand during pregnancy and lactation, menstrual bleeding, and nutritional inadequacy during the reproductive cycle [2].

Moreover, some research [3–5] attempted to test the hypothesis of a link between biomass fuel smoke exposure and anemia. These studies have indicated an association between unclean cooking fuel smoke exposure and anemia in children [5] and pregnant women [3,4]. However, the veracity of their findings remains uncertain and the biological validity of the link between solid fuel and anemia is still being investigated. It is believed that the chemicals released by the combustion of solid fuel can limit oxygen delivery to tissues and produce systemic inflammation, which is mediated by inflammatory cytokines and can disrupt the erythropoietin process, leading to anemia [6–8]. The usage of biomass fuel for day-to-day energy is more common in developing countries than the developed countries, where anemia is also prevalent. It is estimated that about 2.6 billion people worldwide continue to use solid fuels such as wood, animal dung, and agricultural residues for cooking and heating purposes [9]. Incomplete combustion of solid fuel results in the emission of large amounts of particulate matter, carbon monoxide (CO), hydrocarbons, free radicals, and chlorinated organics [10]. In South Asian countries, reproductive-aged women bear a disproportionate share of household cooking responsibilities, putting them at greater risk of being exposed to higher levels of indoor air pollution caused by solid fuel stoves, and this scenario remains unchanged in the Sri Lankan context. However, there has been a paucity of research on the detrimental health effects of biomass smoke exposure in the Sri Lankan context, where most people use firewood exclusively or in combination with liquid petroleum gas (LPG) or kerosene. Thus this study aimed to assess the link between biomass fuel smoke exposure and anemia among non-pregnant, reproductive-aged women in Sri Lanka.

## Materials and methods

### Design, setting, and participants

This study is a part of a larger study that included 403 reproductive-aged women (15–49 years) from Central Province, Sri Lanka. Central province is divided into 48 Medical Officer of Health (MoH) areas, which are clearly defined health units in the community that correspond to the country’s administrative divisions. Public Health Midwife (PHM) areas are the lowest operational unit in the government healthcare system and are located within MoH areas.

**Fig 1:**
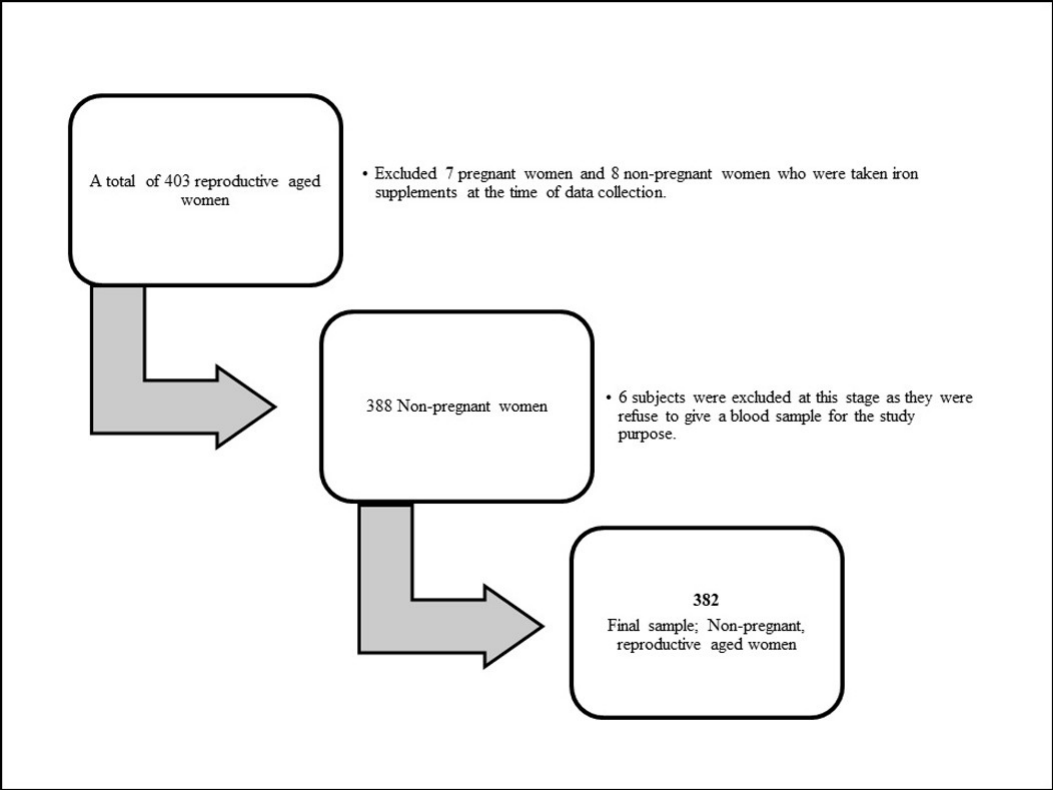
Selection of subjects for the study.

A multi-stage cluster survey was carried out encompassing all three districts in the Central Province, namely Kandy, Matale, and Nuwara Eliya. For the first stage of sampling, eight MoH areas from the Kandy district and four MoH areas each from Matale and Nuwara Eliya districts were chosen using a simple random sampling technique. Then, three PHM areas were picked randomly from each designated MoH area, as the second stage. The number of subjects from each district was determined using the probability proportional to size technique, based on the total population resident in that particular district, according to the data of the fourteenth national Census conducted in 2012 [11]. The total number assigned to each district was then approximately equally distributed among MoH areas. Data were collected beginning at a random point in each selected PHM area and then visiting every second house after that. The study comprised reproductive-aged women with household cooking responsibilities who lived in Central Province, Sri Lanka, from January 2020 to December 2021. Despite the fact that the study was supposed to be completed in a year, it took longer than expected due to the lock-downs and travel restrictions imposed by the Sri Lankan government to mitigate the spread of the COVID-19 pandemic. The main study excluded the women who had previously been diagnosed with heart disease, chronic chest diseases, asthma, or acute or chronic bronchitis. Besides, the women who refused to give blood samples, pregnant women and women who were taken iron supplements were excluded from this part of the study (Fig 1).

### Ethical considerations

The study was approved by the ethics review committee of the Faculty of Allied Health Sciences, University of Peradeniya, Sri Lanka (AHS/ERC/2018/098). The study was conducted in compliance with the principles of the Declaration of Helsinki. Informed written consent was obtained from the participants prior to the data collection. In addition to the participant’s verbal consent, the parent or legal guardian’s informed consent was requested if the eligible participant was under the age of 18.

### Procedure

Data on socio-demographics, economics, cooking fuel use, and exposure to other sources of indoor air pollution such as active or passive tobacco smoke and mosquito coil smoke were collected using a pre-tested structured questionnaire. A hand-held CO monitor called the Micro+TM Smokerlyzer® (Bedfont Scientific Ltd., Maidstone, UK) was used to measure CO concentration and carboxyhemoglobin (COHb) percentage in exhaled air. Micro+TM Smokerlyzer® is a breath CO meter frequently used in clinical trials and smoking cessation programs. Participants were instructed to hold their breath for 15 seconds before blowing into the instrument. Three measures were taken from a single individual, with the highest measurement serving as the final reading. A one-minute pause separated each measurement. Breath CO measurements were performed within three hours following meal preparation, either in the morning or during the day. CO levels in the breath were measured in parts per million (ppm), and COHb levels were approximated as a percentage of oxygen replaced. CO readings of 0–6 ppm and corresponding %COHb levels of 0.00–1.59% were considered to be equivalent to non-smoker levels [12]. The breath CO monitor is accurate to within±2 ppm, and it was calibrated at least every six months, as recommended by the manufacturer. For the purpose of blood hemoglobin concentration measurement, 1.5 ml of venous blood was drawn from each woman after obtaining consent again just before the procedure. The blood samples were collected into EDTA tubes. During the procedure, strict aseptic measurements were undertaken to prevent the possible risk of infections. The collected blood samples were analyzed using Drabkin’s cynomethhemoglobin method to assess the blood hemoglobin in a standard medical laboratory. Safety measures were taken while handling drabkin’s solution as it is a hazardous chemical that contains potassium cyanide. Anemia and non-anemia were defined as hemoglobin concentrations of < 12.0 and ≥ 12.0 g/dL, respectively, based on WHO guidelines for hemoglobin levels, to diagnose anemia for non-pregnant women above 15 years of age [1]. Mild, moderate, and severe anemia were then characterized as hemoglobin concentrations of 11.0–11.9 g/dl, 8.0–10.9 g/dl, and below 8.0 g/dl, respectively [1].

### Statistical analysis

Minitab statistical software (version 20) was used for all statistical analyses (Minitab Inc., State College, PA, USA). The frequency and percentages, as well as the mean and standard deviation (SD) of descriptive statistics, are displayed. To check whether all variables matched the distributional assumptions of the statistical tests used to examine them, they were first assessed using numerical and graphical techniques. The mean differences in hemoglobin among different cooking fuel users (firewood only, LPG only, kerosene only, firewood plus LPG and firewood plus kerosene) were investigated using one-way analysis of variance (ANOVA). Tukey’s post hoc test was used to pairwise comparisons of mean hemoglobin levels following the one-way ANOVA. Individual factors influencing the anemic and non-anemic status of the study population were identified using univariate binary logistic regression analyses. The variables that had a p ≤ 0.3 in the univariate analyses were then chosen for multivariate logistic regression analysis. The individual factors affecting blood hemoglobin concentration were also identified using one-way ANOVA. Following that, the independent variables with p ≤ 0.3 in one-way ANOVA were chosen for multivariate regression analysis. Multicollinearity was determined using the variance inflation factor (VIF), and variables with a VIF < 5 were included in multivariate model. To determine the statistical significance of the related variables, the odds ratio (OR) and 95% confidence interval (CI) were calculated. For each analysis, 95% confidence intervals were calculated, and p < 0.05 was considered statistically significant.

## Results

### Participants’ characteristics

This section of the study comprised 382 non-pregnant women. The participants’ mean age was 37.3 ± 8.9 years. 78.5% were married, and 58.3% were homemakers. More than two-thirds of those who took part in the study had a nuclear family. 23.6% of households reported that they used firewood exclusively for cooking, while 71.5% reported firewood alone or combined with LPG or kerosene (Table 1).

**Table 1:** Characteristics of the study participants.

Variable		Frequency (%)
Age (years) ^‡^		37.3 (8.9)
Civil Status		
	Single	57 (14.9)
	Married	300 (78.5)
	Divorced	13 (3.4)
	Widowed	12 (3.1)
Level of education		
	No school education	5 (1.3)
	Up to primary	40 (10.5)
	Up to ordinary level	176 (46.1)
	Up to advanced level	118 (30.9)
	Higher education	43 (11.2)
Employment		
	Homemaker	223 (58.4)
	Employed	159 (41.6)
Monthly family income (LKR)		
	<14,999	15 (3.9)
	15,000–22,499	55 (14.4)
	22,500–45,999	164 (42.9)
	46,000–149,999	147 (38.5)
	>150,000	1 (0.3)
Family type		
	Nuclear	273 (71.5)
	Extended	109 (28.5)
Cooking fuel type		
	Firewood only	90 (23.6)
	LPG only	108 (28.3)
	Kerosene only	1 (0.3)
	Firewood plus LPG	166 (43.4)
	Firewood plus kerosene	17 (4.4)
Exposure to secondhand tobacco smoke		
	Yes	135 (35.3)
	No	247 (64.7)
Exposure to mosquito coil smoke		
	Yes	87 (22.8)
	No	295 (77.2)
Expired breath CO concentration (ppm) ^‡^		4.1 (4.0)
Percentage of carboxyhemoglobin^‡^		1.3 (0.7)

^‡^ Mean (SD), n = 382, SD: Standard deviation, LKR: Sri Lankan Rupee, LPG: Liquid petroleum gas, CO: Carbon monoxide.

### Status of anemia based on the kitchen fuel type

The study sample’s reported mean blood hemoglobin concentration was 12.2 ± 1.7 g/dl. Of the total sample, 36.1% (n = 138) women were anemic. Mild, moderate, and severe anemia was found in 16.8%, 17.5%, and 1.8% of women, respectively (Table 2). One-way ANOVA revealed no difference in mean hemoglobin concentrations across different cooking fuel type users (Table 3). The difference remained unchanged even between the firewood alone or in combination users and exclusive LPG users (12.2 ± 1.7 g/dl vs. 12.4 ± 1.8 g/dl) (p > 0.05). However, it was found that exclusive LPG users had considerably lower exhaled breath CO levels and estimated %COHb than firewood-only users and firewood plus kerosene users (p < 0.001).

**Table 2:** Status of anemia based on the type of cooking fuel.

Type of cooking fuel	Status of anemia			
	Non-anemic n (%)	Mild anemia n (%)	Moderate anemia n (%)	Severe anemia n (%)
Firewood only; (n = 90)	55 (61.1%)	20 (22.2%)	14 (15.6%)	1 (1.1%)
LPG only; (n = 108)	72 (66.7%)	16 (14.8%)	18 (16.7%)	2 (1.8%)
Kerosene only; (n = 01)	-	-	1 (100.0%)	-
Firewood plus LPG; (n = 166)	105 (63.2%)	25 (15.1%)	32 (19.3%)	4 (2.4%)
Firewood plus kerosene; (n = 17)	12 (70.6%)	3 (17.6%)	2 (11.8%)	-

**Table 3:** Mean hemoglobin concentrations, exhaled breath CO levels and estimated COHb percentages across different cooking fuel type users.

Type of cooking fuel	Blood hemoglobin concentration (g/dl); Mean (SD)	Exhaled breath CO levels (ppm)	Estimated COHb percentages (%)
Firewood only; (n = 90)	12.2 (1.6) ^a^	5.7 (5.1) ^b^	1.5 (0.8) ^e^
LPG only; (n = 108)	12.4 (1.8) ^a^	3.0 (3.05) ^c, d^	1.1 (0.5) ^f, g^
Kerosene only; (n = 1)	9.9 ^a^	9.0 ^b, c, d^	2.1 ^e, f, g^
Firewood plus LPG; (n = 166)	12.0 (1.7) ^a^	3.6 (3.5) ^d^	1.2 (0.6) ^g^
Firewood plus kerosene; (n = 17)	12.8 (1.5) ^a^	7.0 (4.3) ^b^	1.7 (0.7) ^e^

^a, b, c, d, e, f, g^ Values with the same superscript lower case letter do not represent a significance difference. Compared using one-way ANOVA followed by Tukey’s post-hoc test.A,7 b, c tag estimated.

### Binary logistic regression analysis for the status of anemia

Firstly, univariate binary logistic regression analyses were used to identify the factors associated with the anemic status of reproductive-aged women. Univariate analyses were carried out considering anemic status as the dependent variable; maternal age, civil status, level of education, employment, monthly family income, family type, cooking fuel type, the status of exposure to mosquito coil smoke, the status of secondhand tobacco smoke and CO concentration as the independent variables. The variables with a p-value ≤ 0.3 in the univariate analyses were selected (maternal age, family type, cooking fuel type, and status of exposure to mosquito coil smoke) for the multivariate logistic regression analyses. None of the included variable in the multivariate logistic regression model was significant at the level of p < 0.05.

### Multivariate regression analysis for blood hemoglobin concentration

A similar approach to binary logistic regression was used to construct the multivariate regression model for blood hemoglobin concentration. Firstly, univariate analyses (ANOVA) were performed considering the blood hemoglobin concentration as the dependent variable while considering maternal age, civil status, level of education, employment, monthly family income, family type, cooking fuel type, the status of exposure to mosquito coil smoke, the status of secondhand tobacco smoke and breath CO concentration as the independent variables. The variables with a p-value ≤ 0.3 in the univariate analyses (maternal age, employment, family type, cooking fuel type, the status of exposure to mosquito coil smoke, and status of exposure to secondhand tobacco smoke and breath CO concentration) were considered for the multivariate regression model. None of the included factors were significant at the level of p < 0.05.

## Discussion

To the best of our knowledge, this is the first study in Sri Lanka to investigate whether the use of firewood as a cooking fuel is associated with anemia in non-pregnant women compared to other cooking fuel users and its relationship with blood hemoglobin concentrations. Even though many researchers attempt explore a link between solid fuel smoke exposure and anemia, this area is under researched in the Sri Lankan context. The detection of biomarkers in the human body is a helpful tool for quantifying exposure to chemicals or mixtures of chemicals, as well as early biological impacts as a result of exposure [13]. Firewood smoke contains several contaminants, including CO, which has the potential to cause systemic inflammation [14]. Systemic inflammation, mediated by inflammatory cytokines such as tumor necrosis factor-alpha (TNF-α), interleukin-1 (IL-1), interleukin-6 (IL-6), and interferon- (IFN- γ), is a well-known cause of anemia [7]. Thus the current study also examined the levels of breath CO and %COHb in participants, which are unique biomarkers of CO exposure for humans. However, the expired breath CO levels indicate not only the intensity of exposure to cooking fuel smoke but also the exposure to other air pollutants like exhausted vehicle fumes, cigarette smoke, and mosquito coil smoke. In the current study, the levels of breath CO and %COHb were taken directly from the readings of Micro+TM Smokerlyzer®, where %COHb reflects only an estimate based on the CO readings, which is converted to %COHb using a calibration curve through the machine itself. Among the few studies that have investigated the relationship between CO exposure and exhaled breath CO and %COHb in women, Rudra et al., 2010 revealed a low mean %COHb (0.93 ± 0.43) among pregnant women [15] compared to the non-pregnant women in the current study (1.3 ± 0.7). The difference in %COHb levels in the two studies might be attributable to the self-protective measures against smoke exposure during pregnancy. However, the study done by Rudra et al. was based on blood sample analysis to assess the %COHb, which makes it challenging to make a direct comparison with our study. Moreover, the current study found no correlation between the two factors; personal breath CO levels and blood hemoglobin concentrations. Although no previous studies have been undertaken to analyze the impact of biomass fuel smoke on anemia in non-pregnant women, few studies have been conducted to assess the same in pregnant women [3,4] and children [5]. In the current study, one household used kerosene as the primary cooking fuel. After excluding the kerosene user, the remaining data revealed that the firewood plus LPG users had the lowest mean blood hemoglobin concentration, but were not significantly different from other types of cooking fuel users. Moreover, no difference in the prevalence of anemia was found based on the type of cooking fuel. In contrast, studies done in Sub-Saharan Africa [5], India [3], and Ethiopia [4] found an increased risk of anemia among children and pregnant women when they are exposed to solid fuels. However, the differences in populations and settings made it difficult to do a fair comparison.

Nevertheless, the current study did not examine the dietary intake and the nutritional status of the participants, which can significantly influence the hemoglobin status of women. More than 50% of the participants in this study were under the mean average national household income reported for the year 2016 [16], and it might reflect the current study’s finding of the high prevalence of anemia and the vulnerability of anemia among reproductive-aged women in this study population. Conversely to the findings of studies done in Sub-Saharan Africa [5], India [3], and Ethiopia [4], some researchers revealed increased hematocrit, hemoglobin levels and red blood cell counts among smokers [17,18]. The elevated levels of hematological parameters in smokers believed to be a compensatory mechanism against the decreased oxygen delivering capacity [19]. In the current study, there were no single active smoker, but 35.3% reported exposure to secondhand tobacco smoke. Also, 71.5% of households had been used firewood exclusively or in combination with kerosene or LPG, which can lead to detrimental health effects among those exposed, especially respiratory-related diseases. However, the use of cleaner fuel in Sri Lanka is largely hindered by its cost and the free availability, and easy access to firewood.

This study has several limitations. Firstly, the cross-sectional study design merely allows for investigating the connection between predictors and outcome variables, not a causal relationship. Secondly, the results were not adjusted for the nutritional status of participants. Additionally, using Drabkin’s cynomethhemoglobin method to determine blood hemoglobin concentrations may result in incorrect hemoglobin values, as abnormal plasma proteins and high leukocyte count can cause turbidity in diluted blood. Despite the aforementioned limitations, to the best of our knowledge, this is the first study in Sri Lanka conducted at the provincial level to assess the relationship between cooking fuel usage and anemia among reproductive-aged women. Furthermore, this study also assesses individuals’ expired breath CO levels and their association to the blood hemoglobin concentration.

## Conclusions

The study results suggest no significant effect of solid fuel smoke exposure on the anemic or non-anemic status of non-pregnant reproductive-aged Sri Lankan women. However, the findings should be used with caution due to the study’s cross-sectional nature, and further larger-scale prospective cohort studies are recommended. The reasons behind the high prevalence of anemia among reproductive-aged women should be ruled out, and corrective measures should be implemented urgently. Further studies on testing the link between biomass fuel smoke exposure and cytokines and other biomarkers that may influence the anemic or non-anemic status or the process of erythropoiesis, are recommended.
